# Comment on: Male reproductive aging: can men with oligospermia become azoospermic over time?

**DOI:** 10.1038/s41443-024-00985-5

**Published:** 2024-10-08

**Authors:** Gilad Karavani

**Affiliations:** Infertility and IVF Unit, Department of Obstetrics and Gynecology, Hadassah Ein-Kerem Medical Center and Faculty of Medicine, Hebrew University of Jerusalem, Jerusalem, Israel

**Keywords:** Medical research, Risk factors

In a recently published article, Patel et al. addressed the pressing issue of male reproductive aging, particularly focusing on whether men with oligospermia (sperm count <15 million sperm/mL) are at risk of progressing to nonobstructive azoospermia (NOA) [1]. They provide insights into the causes, including aging, genetic defects, and lifestyle factors. Patel et al. offer valuable recommendations on managing this progression, which are crucial for clinicians treating male infertility.

The authors emphasize the current limitations of semen analysis, which provides a cross-sectional view of a man’s semen quality status but fails to predict future reproductive potential. This poses a particular challenge when dealing with patients with severe oligospermia. Patel et al. highlight a study by Song et al. [2], which followed men with severe oligospermia over a median period of 3.5 years, discovering that about 13% of these men progressed to NOA. Additionally, the study revealed that some men experienced a significant decline in sperm count, with some developing cryptozoospermia (sperm detectable only after centrifugation). This progression, though affecting a small proportion of men, raises critical concerns about reproductive aging that warrant further investigation. The same group reported comparable findings in a larger study that categorized patients based on the severity of oligospermia [3].

The authors draw from these studies and their clinical experience, noting that while the majority of oligospermic men maintain stable semen parameters, a smaller group experience a decline over time. This phenomenon of progressive spermatogenic failure suggests the potential involvement of underlying genetic defects or environmental factors that drive men past a critical threshold. Despite the lack of a clear understanding of the exact mechanisms, it highlights the importance of proactively monitoring these patients and offering counseling on their fertility options.

One additional point discussed in this review is the relationship between aging and sperm production. Unlike female fertility, which is well understood to decline with age, the effect of aging on male fertility is less straightforward. Patel et al. present conflicting data, with some studies showing a decline in some semen parameters, while others find no significant change as men age [4–6]. The authors elaborate on the hypothesis that reserve spermatogonia may be over-recruited to compensate for lower spermatogenic efficiency [7]. While this process maintains sperm production initially, it may eventually deplete the pool of stem cells, leading to a further decline in spermatogenesis [8]. This depletion of stem cells may explain why some men with severe oligospermia progress to azoospermia, while others remain stable. However, though this hypothesis is compelling, the authors acknowledge that further research is needed to confirm whether over-recruitment of reserve spermatogonia is indeed responsible for the observed decline in sperm production in older men. The authors suggest that identifying men at risk for stem cell depletion could help predict which patients are most likely to experience a further decline in their reproductive potential.

Finally, the authors also underscore the importance of addressing modifiable risk factors. Lifestyle changes such as smoking cessation, weight loss, and avoiding environmental toxins like endocrine-disrupting chemicals (EDCs) could potentially slow the decline in sperm production [9]. The authors recommend that patients be educated about these risk factors, especially young men diagnosed with severe oligospermia, as early intervention may help preserve their reproductive potential.

In light of the findings discussed, Patel et al. propose several practical recommendations for clinicians managing men with severe oligospermia. First, they advocate for regular follow-up, recommending semen analysis every one to two years to monitor any decline in sperm count. This approach allows for the early detection of fertility deterioration and offers an opportunity for patients to take proactive measures before their condition worsens. Second, they suggest that men with severe oligospermia should be counseled about the possibility of declining sperm parameters and offered the option of sperm cryopreservation. This strategy, commonly used for men undergoing gonadotoxic treatments such as chemotherapy, could be extended to oligospermic men to preserve their fertility for future use.

A more recent paper by Karavani et al. [10] which retrospectively analyzed a large cohort of over 1000 men with different degrees of oligospermia with time, aligns with their observations and most importantly, quantifies the risk of secondary non-obstructive azoospermia, particularly in men with extremely low sperm concentrations (<1 million/mL). These findings reveal that the incidence of secondary azoospermia is significantly higher in men with an initial sperm concentration below 1 million/mL, with rates increasing over time, compared to those with milder forms of oligospermia (1–5 million/mL and above 15 million/mL). Specifically, these rates rose from 5.4% during the first year to 32.0% in men who were followed for more than five years. These results underscore the importance of early sperm banking for men with severe oligospermia of less than 1 million/mL, a recommendation that complements the perspective provided by Patel et al. Furthermore, this study identified lower testosterone levels as a predictive factor for secondary azoospermia in these men, while follicle-stimulating hormone (FSH) and luteinizing hormone (LH) levels did not show the same association. This insight adds another layer to the understanding of male infertility scarcely investigated, suggesting that hormone levels, particularly testosterone, should be closely monitored in men at risk of developing azoospermia.

This data from Karavani et al. contributes additional evidence that supports the authors conclusions and emphasizes the importance of early and proactive fertility preservation strategies (sperm banking) for men with severe oligospermia. Together, these insights can guide clinicians in offering more personalized and effective care for men facing the risk of developing non-obstructive azoospermia.
